# An epigenome‐wide study of a needs‐based family intervention for offspring of trauma‐exposed mothers in Kosovo

**DOI:** 10.1002/brb3.70029

**Published:** 2024-09-11

**Authors:** Joanne Ryan, Aung Zaw Zaw Phyo, Sebahate Pacolli Krasniqi, Selvi Izeti Carkaxhiu, Peter Fransquet, Sara Helene Kaas‐Petersen, Dafina Arifaj Limani, Vjosa Devaja Xhemaili, Mimoza Salihu, Qendresa Prapashtica, Nebahate Zekaj, Vesa Turjaka, Shr‐Jie Wang, Feride Rushiti, Line Hjort

**Affiliations:** ^1^ Biological Neuropsychiatry and Dementia Unit, School of Public Health and Preventative Medicine Monash University Melbourne Australia; ^2^ Kosovo Rehabilitation Center for Torture Victims (KRCT) Pristina Kosovo Australia; ^3^ Faculty of Health, School of Psychology, Centre for Social & Early Emotional Development Deakin University Geelong Victoria Australia; ^4^ The Danish Institute Against Torture (DIGNITY) Copenhagen Denmark; ^5^ Novo Nordisk Foundation Center for Basic Metabolic Research, Metabolic Epigenetics Group, Faculty of Health and Medical Sciences University of Copenhagen Copenhagen Denmark; ^6^ Department of Obstetrics, Center for Pregnant Women with Diabetes Copenhagen University Hospital Copenhagen Denmark

**Keywords:** DNA methylation, intergenerational, intervention, trauma

## Abstract

**Introduction:**

Maternal stress and trauma during pregnancy have been shown to influence cortisol levels and epigenetic patterns, including DNA methylation, in the offspring. This study aimed to determine whether a tailor‐made family intervention could help reduce cortisol levels in children born to traumatized mothers, and to determine whether it effected offspring DNA methylation. The secondary aim was to determine whether the family intervention influenced DNA methylation aging, a marker of biological aging.

**Methods:**

A needs‐based family intervention was designed to help address relational difficulties and family functioning, and included a focus on family strengths and problem‐solving patterns. Women survivors of sexual violence during the Kosovar war in 1998–1999, and their families (children with or without partners) were randomly assigned to 10 sessions of a family therapy over a 3–5‐month period, or to a waitlist control group. Both mothers and children completed assessments prior to and after the intervention phase. Children's blood samples collected at these two time points were used to measure cortisol and epigenome‐wide DNA methylation patterns (Illumina EPIC array). Cortisol levels, and genome‐wide DNA methylation changes pre‐/postintervention were compared between children in the intervention and the waitlist groups. DNA methylation age and accelerated biological aging were calculated.

**Results:**

Sixty‐two women–child dyads completed the study, 30 were assigned first to the intervention group, and 32 to the waitlist control group. In adjusted linear regression, the family intervention was associated with a significant decline in cortisol levels compared to the waitlist control (*β* = −124.72, 95% confidence interval [CI]: −197.4 to −52.1, *p* = .001). Children in the intervention group, compared to the waitlist control group, showed >1% differential methylation degree at 5819 CpG (5'—C—phosphate—G—3') sites across the genome (*p* < .01), with the largest methylation difference being 21%. However, none of these differences reached genome‐wide significant levels. There was no significant difference in DNA methylation aging between the two groups.

**Conclusion:**

We find evidence that a tailored family‐based intervention reduced stress levels in the children (based on cortisol levels), and modified DNA methylation levels at a number of sites across the genome. This study provides some preliminary evidence to suggest the potential for tailored interventions to help break the intergenerational transmission of trauma, however, large studies powered to detect associations at genome‐wide significant levels are needed.

## Graphical abstract

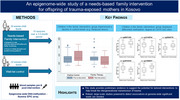

## 1 INTRODUCTION

Severe stress and trauma are important risk factors implicated in a number of psychopathologies (Hogg et al., 2023). Depression and anxiety can commonly occur following major stress/trauma (Chu et al., 2013). Most directly linked is post‐traumatic stress disorder (PTSD), which can result from a severe traumatic event and is characterized by re‐experiencing symptoms, hyperarousal, avoidance, and negative cognition and mood (American Psychiatric Association, 2013). The longer term impacts of trauma, however, can be more widespread, impacting not only mental but also physical health (McFarlane, 2010).

There is now increasing evidence to suggest that the effects might also impact future generations (Yehuda & Lehrner, 2018; Zhou & Ryan, 2023). A number of studies have shown that stress and trauma during pregnancy can influence the health of the offspring (Amici et al., 2022; Zietlow et al., 2019). This could include dysregulation of their stress response system, neurodevelopmental delays, and an increased risk of psychiatric conditions (Coussons‐Read, 2013; Goldstein et al., 2021).

Epigenetics are one proposed mechanism which could account for the biological embedding of severe stress and trauma (Ryan et al., 2016). Epigenetics are environmental sensitive modifications to the DNA which can influence gene expression and function. There is now good evidence to show that the prenatal in utero environment is involved in shaping the epigenome of the developing fetus, and influence the offspring's future health. For example, maternal smoking during pregnancy is associated with decreased DNA methylation at the *AHRR* gene in offspring, and this remains stable postnatally (Joubert et al., 2016). AHRR methylation has also been shown to mediate the link between offspring birthweight and anthropometrics (Küpers et al., 2015). Other diverse exposures in utero have also been found to modify the offspring's epigenetic profile. This includes maternal alcohol consumption (Oei, 2020), nutrition (Di Costanzo et al., 2022), diabetes (Hjort et al., 2019), air pollution (Johnson et al., 2021), as well as maternal mental well‐being (Ryan et al., 2017), and major trauma (Perroud et al., 2014; Radtke et al., 2011).

Trauma has also been associated with accelerated DNA methylation aging, a marker of biological age (Ryan et al., 2020). Findings from a meta‐analysis showed that childhood trauma and PTSD severity were positively associated with DNA methylation aging (Wolf et al., 2018). A recent study of maternal adverse childhood experiences found that mother's reporting ACEs prior to pregnancy, had offspring with accelerated biological aging (Nwanaji‐Enwerem et al., 2021). This suggests the possibility that trauma in one generation could potentially leave a legacy effect on the next generation, and this may occur through accelerated aging.

In line with these findings, we have previously shown that children born to women with pregnancy PTSD, who were survivors of sexual violence during the war, had higher cortisol levels than women survivors without pregnancy PTSD (Hjort et al., 2021). Furthermore, we demonstrated differential DNA methylation was observed at a number of genes involved in stress signaling pathways, when comparing the offspring of mother's with and without pregnancy PTSD. DNA methylation of some of these genes was also associated with children's cortisol levels (Fransquet et al., 2022). Epigenetic aging was not investigated.

Counseling and psychological interventions are a first‐line approach for individuals who have experienced trauma (Hoppen et al., 2024; Mavranezouli et al., 2020). Family‐based therapies have been shown to be effective to address a wide range of issues within a family, including the impact of trauma (Fictorie et al., 2022; Oberg & Sharma, 2023). An important unanswered question, however, is whether therapies which help address the impact of intergenerational trauma, could also result in changes in DNA methylation patterns (Zhou & Ryan, 2023). As a first step to addressing this question, we assessed the needs of women survivors of sexual violence during the war and their families, and developed a family‐based intervention specifically tailored to these needs. We then randomized mothers and their families (children/s with or without partners) to this intervention, which was delivered as 10 sessions over a 3–5‐month period, or to a waitlist control group.

The primary aims of this current study were to (i) determine whether children receiving the family‐based therapy had a reduction in cortisol levels compared to children in the waitlist control group; and (ii) determine whether this therapy had an effect on children's DNA methylation patterns. The secondary aim was to determine whether the family therapy influenced DNA methylation aging, a marker of biological aging.

## 2 METHODS

### 2.1 Participants

The Kosovo Rehabilitation Centre for Torture (KRCT) is an independent, nongovernmental and not‐profit organization that was founded in 1999 in response to the need for psychosocial support of torture and trauma survivors from the Kosovo war (1998–1999; Lopes Cardozo et al., 2000; Wenzel et al., 2009). Our previous study investigating the association between maternal PTSD during pregnancy and offspring DNA methylation (Hjort et al., 2021), included women who were registered in the KRCT database as survivors of sexual violence during the war in Kosovo, and were born in Kosovo, lived there during the war in 1998–1999 and were of Albanian ethnicity. They also needed to have given birth to at least one child after the end of the war. Excluded, however, were mothers and/or children with major health conditions: mental or growth retardation, significant speech or cognitive impairment, major alcohol or substance abuse, schizophrenia or recent chemotherapy/radiotherapy. The 117 mothers who participated in that study (Hjort et al., 2021) were eligible for inclusion in this current study. All women had a lifetime history of PTSD (diagnosed based on DSM‐IV criteria) and a large proportion reported PTSD symptoms during pregnancy. Women willing and able to take part in the study examinations at KRCT with at least one of their children, and who agreed to provide blood samples, were involved in the current study.

### 2.2 Study design

In February 2021, we assessed mothers and their families (children and/or partners) to obtain information on their sociodemographics, lifestyle, and environment, as well as any specific needs. This information informed the development of a family‐based intervention to help address relational difficulties and family functioning in the home and included a focus on family strengths and problem‐solving patterns. The families were randomly allocated to one of three KRCT therapists by a block randomization procedure using a computerized random number generator by three blocks of size 32, created by staff from the Danish Institute Against Torture (DIGNITY) who were not involved in the trial. Each participant was given a unique number. The participants in each group were then randomly assigned to the intervention group or the waiting list group, using a block randomization technique performed by the same DIGNITY staff not involved in the trial.

After the baseline assessments and bloods were taken, mothers and their family members were together randomly assigned to 10 sessions of a family therapy (adapted from the UK Association for Family Therapy and Systemic Practice; Association for Family Therapy and Systemic Practice) over a 3–5‐month period, or to the waitlist control group, commencing in September 2021 and continuing into January 2022. Mothers were administered questionnaires and clinical assessments: Albanian version of Strength and Difficulties Questionnaire (SDQ) and the Systemic Clinical Outcome and Routine Evaluation‐15 (SCORE‐15) were conducted, height, weight, mid–upper arm circumference, blood pressure, pulse were measured at baseline and postintervention (6 months later). Bloods were also collected at these time points.

### 2.3 Intervention

The Family Therapy and Systemic Practice program was designed and implemented by KRCT. The purpose of the program is to reduce the risk of intergenerational transmission of trauma in families whose mothers are survivors of wartime sexual violence. A UK‐based certified trainer of the UK Association of Family Therapy and Systemic Practice and whose mother tongue was Albanian, was a consultant for the project, and developed a training curriculum in the Albanian language to train the staff at KRCT. The curriculum addressed the links between systemic and other related approaches, and the transfer of theory into practice supported by exercises, videos, and discussions of KRCT cases. The program covered all aspects from engaging families in systemic communication and change, as well as instructions and reflections on how to end the therapy. The curriculum included crucial generic elements of systemic theory and practice, as well as a focus on the impact of trauma in the attachment relationship.

All mothers who participated in the study had received individual trauma‐focused therapy at KRCT previously, and thus the therapists delivering the intervention at KRCT had an established connection with the mother. This motivated mothers to participate in the study and attend all sessions with their families. Problems were defined with the families at intake, but other problems emerged during family therapy. The family dynamic was mapped systematically, and therapists made systemic hypotheses that guided the intervention. Ten sessions were run with the families over a 3–5‐month period. Families received follow‐up phone calls by the family therapists 1 month after the family therapies were completed, and were offered additional session if needed. Individuals in the waitlist control group received the intervention 6 months later. Regardless of the group assignment, all mothers were able to continue accessing and receiving individual trauma‐focused therapy, as they had prior to enrolment in the current study. Information on whether mothers had or had not participated in such counseling, was included as a covariate in the analysis.

### 2.4 Blood collection and cortisol assay

Fasting peripheral blood samples were collected from children between 7.30 am and 9.30 am, at the Tirana Laboratory in Pristina, Kosovo within 1 month prior to the start of the intervention, and again at the end of the intervention. The laboratory was completely independent and separate to the clinical center where the family therapy sessions took place. Blood was collected into a 6 mL EDTA‐coated tube (Vacuette, Hettish Labinstrument ApS), and centrifuged within 1–2 h of collection, separated into plasma and buffy coat aliquots, and then stored at −80°C for later DNA extraction from the buffy coats (see below). Another 6 mL sample was collected in a SARSTEDT tube for cortisol measurement using the electrochemical luminescence immunoassay (Roche).

### 2.5 DNA methylation

DNA was extracted from the white blood cell enriched buffy coats using the DNeasy 96 Blood & Tissue Kit (Qiagen), according to manufacturer's protocols. Extracted DNA was randomly plated by technicians blinded to the randomization status of the participants, and run on the Infinium HumanMethylationEPIC BeadChip array (Illumina) according to the manufacturers protocol at GenomeScan. The programming platform “R” version 4.3.0 with R package *minfi* (Aryee et al., 2014) was used for the preprocessing and quality control of the data according to the Maksimovic pipeline (Maksimovic et al., 2016). The EPIC data were normalized using the subset quantile normalization (SQN, “preprocessQuantile”) method (Touleimat & Tost, 2012). Sample sex was checked as concordant with biological sex using “getSex” of the minfi R package. Probes containing detection *p* values >.01, known single nucleotide polymorphisms (SNPs) probes, sex chromosome probes, and cross‐reactive probes (Pidsley et al., 2016), were removed from the dataset (Supporting Information Table S1). After quality control and data cleaning, a total of 766,130 probes were available for further analysis. The methylation status of each probe was transformed into “*M* values” (log2 of array intensities at each probe) and “Beta values” (average DNAm for each probe as a measure ranging from 0 [unmethylated] to 1 [100% methylated]). The cell counts from the methylation data were estimated using “estimateCellCounts2” (R package *FlowSorted.Blood.EPIC*; Salas et al., 2018).

Biological age and subsequently biological aging were calculated from DNA methylation data, both pre‐ and postintervention. The EPIC data were normalized using the preprocessNoob method and the “DNAm age online calculator” https://dnamage.clockfoundation.org was used. This generated age estimates for Horvath (Horvath, 2013) and Horvath Skin Blood Clock (DNAmAgeSkinBloodClock; Horvath et al., 2018), as well as age acceleration as the residual from regression chronological age on these DNAm aging measures. While the field of epigenetic clocks is continually advancing, these epigenetic clocks were chosen because they have been widely used and are recommend for pediatric bloods samples (Fang et al., 2023; Wang & Zhou, 2021).

### 2.6 Statistical analysis

Included in this current study were data and measures pertaining to the mother and her youngest child. In the case of twin pairs, for whom there were two, the last registered child was excluded from the data analysis. R statistical software and STATA statistical software version 18.0 were used for the analysis.

The change in cortisol levels in children between the intervention and waitlist control groups was examined using linear regression, adjusted for potential confounding factors such as child's sex, and age, pregnancy PTSD, and smoking status, whether mothers received individual therapy, and baseline cortisol levels.

For the analysis of epigenome‐wide DNA methylation differences, a number of steps were taken. First, using the *WGCNA* package, a principal component analysis (PCA) was conducted on the converted *M*‐values, to determine the major sources of variation within the methylation dataset. Considered in this PCA were child age, child sex, child living status, child birthweight, mother's smoking status during pregnancy, mother's PTSD status during pregnancy, individual therapy, and estimated cell counts (CD8T, CD4T, NK, Bcell, Mono, and Neu). The R package “*limma*” linear regression model (Ritchie et al., 2015) adjusting for possible confounding variables was then used to identify the differentially methylated probes (DMPs) between intervention and control groups. The model included intervention status, child age, child sex, child living status, and cell counts (CD8T, CD4T, NK, Bcell, and Neu). Observations were adjusted for false discovery rate by the Benjamini and Hochberg (BH) method. For the top DMPs, linear regression analysis was then performed adjusting for potential confounding variables. This included child's sex, and age, pregnancy PTSD, and smoking status, whether mothers received individual therapy, batch effects, and cell counts, as well as baseline levels of these probes. All CpG genomic locations are described with reference to the GRCh37 (hg19) genome assembly.

In addition to investigating DNA methylation differences at individual CpG sites, we then also examined whether there were differentially methylated regions (DMRs) of interest, between control and intervention groups postintervention. DMRs are defined as ≥2 differentially methylated CpG within 400 bp, and an average effect size of at least 1% methylation difference across the region. The *DMRcate* package was used (Peters et al., 2015), and it ranks DMRs by Stouffer's test statistic.

The change in biological aging from baseline to 6 months (6‐month baseline) was compared between the intervention and control groups using *t*‐tests, and then with adjustment for child's sex, and age, pregnancy PTSD, and smoking status, whether mothers received individual therapy, cell counts, and baseline biological aging using linear regression.

## 3 RESULTS

### 3.1 Participant characteristics

There were 64 women with 66 children (including two twin pairs) who agreed to participate in the Needs Assessment; 31 were randomized to the intervention and 33 to the waitlist control. Of these, two families withdrew consent from the study, one from each group. Given that only one (the first registered) child from the twin pairs was included in the analysis, this left 62 women–child dyads, 30 in the family intervention group and 32 in the waitlist control. In the intervention group, 93% completed the intended 10 family therapy sessions, with two families completing nine of 10 sessions.

Characteristics of the mothers and their children included in this analysis are shown in Table 1. Children in the two groups were similar in terms of age, sex, birthweight, and area in which they lived. Mothers randomized to the family therapy group were significantly less likely to have PTSD during pregnancy (*p* = .04), and were less likely to be smokers (*p* = .02). There was also a significant difference between the intervention and control groups in terms of mothers who received individual counseling. Mothers in the intervention group were significant less likely to seek (and thus receive) individual counseling (*p* = .003).

### 3.2 Cortisol levels

**FIGURE 1:**
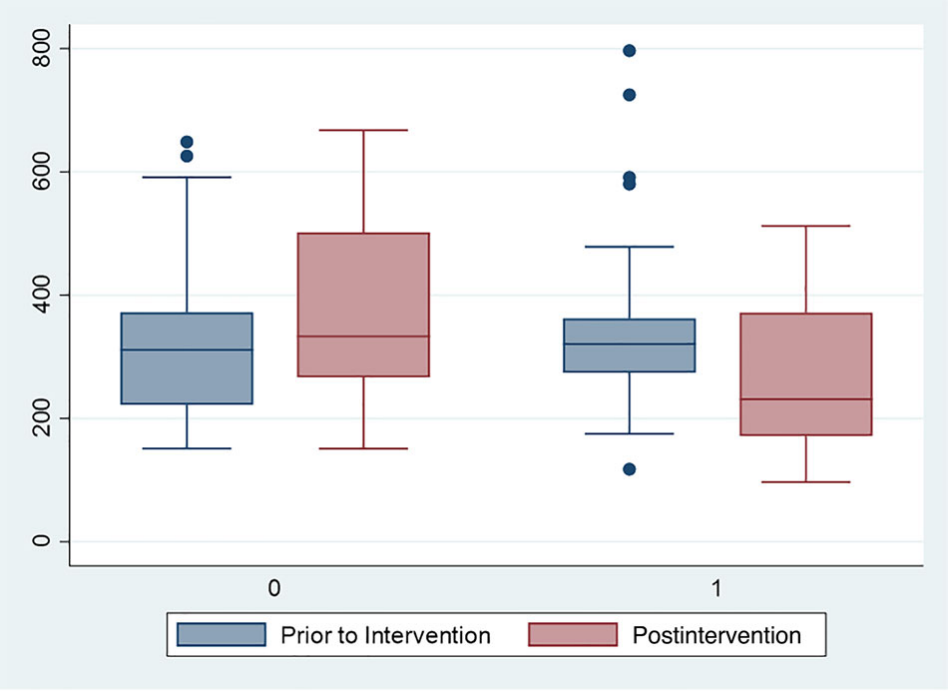
Cortisol levels in children pre‐ and post‐6‐month intervention, for the children who received family therapy (1), and those in the waitlist control group (0).

Children in the family intervention group experienced a significant decline in cortisol levels pre‐ to post‐treatment (Δ 82.3 nmol/L, *p* = .02), whereas no significant decline was observed in the waitlist control group (*p* = .11), and their cortisol levels actually increased slightly over this period (**Figure** **1**). The change in cortisol levels was significantly different between the two groups (*p* = .004). In linear regression analysis, the family‐based therapy group had significantly lower cortisol levels than the waitlist control, even after controlling for sex and age of the child, pregnancy PTSD, maternal smoking during pregnancy, and individual therapy sessions, as well as cortisol levels prior to the intervention (*β* = −124.72, 95% confidence interval [CI]: −197.4 to −52.1, *p* = .001, Supporting Information Table 2). In additional analysis, we also examined whether there was a difference in cortisol levels within the intervention group, based on the specific therapist providing the intervention. No significant differences were found (*p* = .34).

### 3.3 DNA methylation analysis

A total of 5819 differentially methylated CpG sites (DMPs) were identified with a mean difference in children's DNA methylation of >1.0% between the intervention and control groups (raw *p*‐value <.01). Among these 2931 (50.37%) showed higher DNA methylation in the family‐based intervention compared to the waitlist control group. The largest mean methylation difference between groups was 21% for cg15355235 (near the CELF4 gene).

**FIGURE 2:**
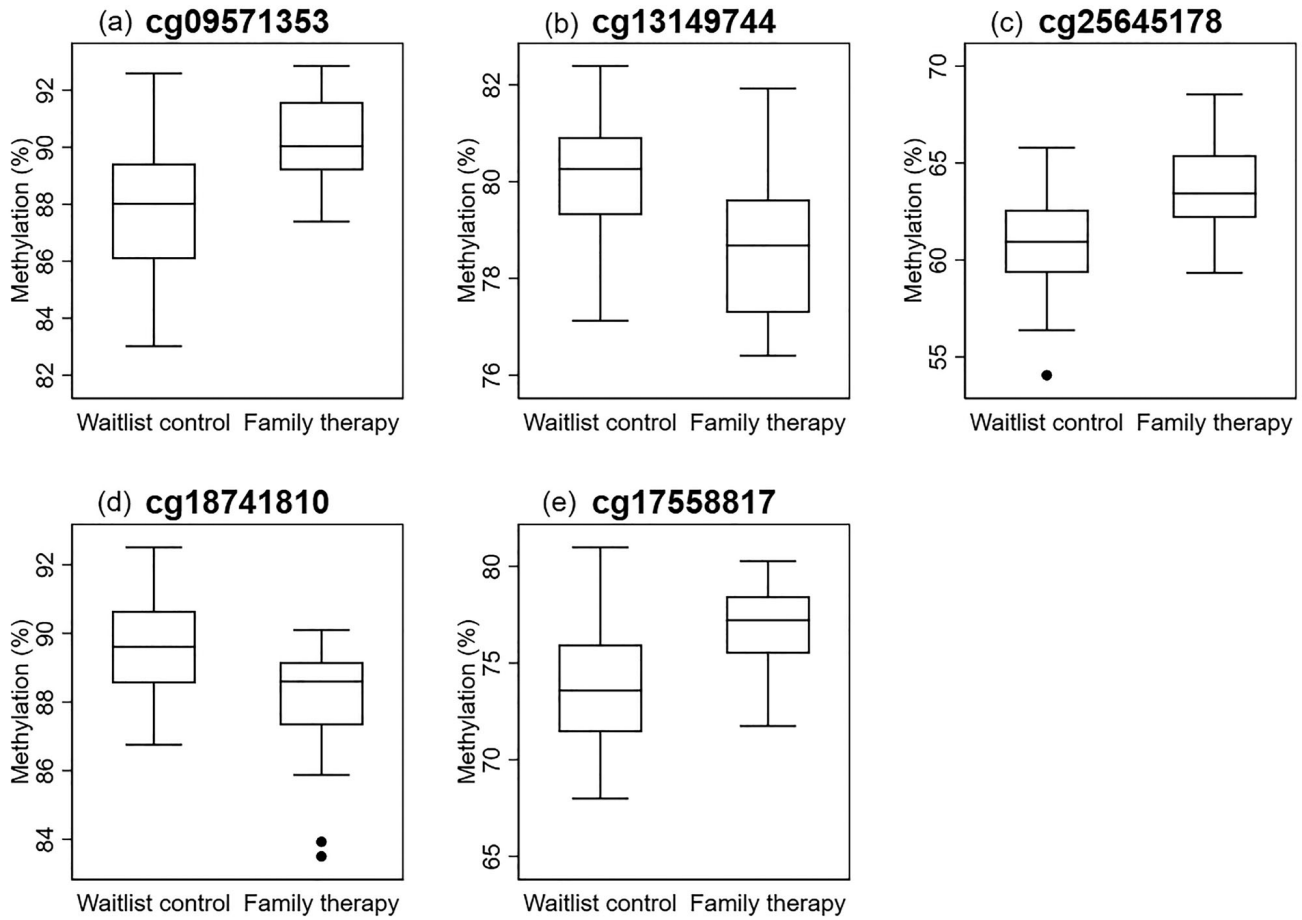
Differential probes comparing methylation of family therapy (*n* = 30) versus waitlist controls (*n* = 32); (a) cg09571353, delta *β*: +2.33%, standard error (SE): 0.48, *p*‐value <.001; (b) cg13149744, delta *β*: −1.58 %, SE: 0.34, *p*‐value <.001; (c) cg25645178, delta *β*: +2.63 %, SE: 0.61, *p*‐value <.001; (d) cg18741810, delta *β*: −1.53%, SE: 0.39, *p*‐value <.001; (e) cg17558817, delta *β*: +3.31%, SE: 0.65, *p*‐value <.001.

The full list of these probes significant at *p* < .01 is shown in Supporting Information Table 3, but none of the probes reached strict significance levels after adjustment for multiple testing. The top 10 DMPs ranked by *p*‐value and with methylation differences of greater than 1% are shown in Table 2. For these probes, linear regression models were run adjusting for a range of covariates, including children's sex, age and birthweight, living area, mother's smoking, and PTSD status during pregnancy, whether mothers received individual therapy and baseline (preintervention) methylation status, as well as batch effects and blood cell composition. Five of the probes remained significantly different between the family therapy and control groups, and these are shown in **Figure** **2**.

A total of 57 DMRs were identified at *p* < .01, based on our criteria of covering at least three CpGs and the average effect size across the region of >1% methylation (Supporting Information Table 4). These DMRs included between three and 43 probes and the regions were between 243 and 2702 base pairs long. The largest average DNA methylation for a DMR was for gene *NHSL1* with five CpGs and an average methylation difference of 9.3%. The top 10 DMR are shown in Table 3. However, none of the DMRs reached statistical significance according to the Stouffer adjusted *p*‐values. As such, pathway analysis was not conducted.

### 3.4 Biological aging

There was a strong correlation between the chronological age of children and their DNAm age (*r* ^2^ = 0.87 for Horvath; 0.92 for Horvath Skin and Blood). DNAm age and age acceleration were not different between children in the two groups at baseline, nor at the second time point, postintervention (Supporting Information Table 5). The change in DNAm age over time was also not significantly different between the intervention and waitlist control groups (Supporting Information Table 6). The results remained consistent after further adjustment (Supporting Information Table 7).

## 4 DISCUSSION

We conducted a study of a 10‐session family needs‐based intervention to women survivors of sexual violence during the war, and their children. We demonstrated that children in the group randomized to the family therapy had a significant reduction in cortisol levels, compared to children in the waitlist control group. We also found that a number of genes and gene regions were differentially methylated between the family therapy and waiting list control groups, however, none of these associations remained significant at genome‐wide corrected levels. Furthermore, we found no significant difference in DNAm age acceleration between children in the two groups.

Trauma can result in disruptions in the hypothalamic–pituitary–adrenal (HPA) axis signaling (Suzuki et al., 2014), and cortisol is the principal glucocorticoid stress hormone which is secreted from the adrenal cortex in response to stress. Cortisol is often used as a marker of stress reactivity, and prior studies have linked cortisol levels with trauma and severe stress in adults and children (Cay et al., 2018; Meuret et al., 2015; Zajkowska et al., 2022). Many, but not all studies, suggest that children exposed to trauma have higher morning cortisol levels, and these are associated with poorer health outcomes, compared to nontrauma‐exposed children (Cicchetti & Rogosch, 2001; Doom et al., 2014). A study found that preadolescent trauma‐exposed children with high cortisol levels had poorer executive function and emotional regulation (Motsan et al., 2022). Likewise, maltreated children with internalizing problems have consistently been shown to have elevated basal cortisol levels in the morning (Tarullo & Gunnar, 2006). Furthermore, the security of the infant–mother attachment has been shown to be associated with cortisol secretion (de Mendonça Filho et al., 2022; Kuo et al., 2019).

We have previously shown that offspring of mothers with prenatal PTSD had higher cortisol levels than offspring born to mothers without PTSD (Fransquet et al., 2022). Our current findings add to this by demonstrating that a family‐based intervention was associated with a significant reduction in cortisol levels compared to the waitlist control group. Although these are preliminary findings and further work is needed to see whether these effects persist longer term, this study highlights the potential that interventions may help mitigate the intergenerational transmission of trauma. In our study, parenting was systematically supported by family therapists. Planning and managing everyday life were an important part of the family therapy and helped to strengthen the bonding and minimize conflict among family members.

When comparing DNA methylation patterns between children in the intervention and control groups, a number of nominally significant differences were observed even after adjusting for potentially important confounding factors, such as age, maternal smoking, and PTSD status, batch effects and cell counts, as well as baseline methylation levels. In particular, increased methylation of cg09571353 in the NFKB Inhibitor Ras Like 1 (*NKIRAS1*) gene, cg17558817 in the NCK adaptor protein 2 (*NCK2*) gene, and cg25645178 in the histocompatibility minor 13 antigen (*HM13*) gene was observed in children who participated in the family therapy, compared to the waitlist control. *HM13* has been involved in genomic imprinting, and is thought to play an essential role in fetal development. Differential DNA methylation in this gene has also been implicated in intrauterine growth restriction (Monteagudo‐Sánchez et al., 2019) and placental stress in pregnancy (Lambertini et al., 2019). Furthermore, a study of 602 adults with a severe mental illness found that differential methylation of the *HM13* gene was associated with reporting of childhood trauma, and sexual abuse specifically (Løkhammer et al., 2022). This finding is of interest in the context of our study, which investigated the potential benefit of an intervention for offspring of women exposed to sexual abuse during the war. Our study also found the offspring in the intervention group had decreased methylation at cg13149744 of Ankyrin Repeat Domain (*ANKRD20B*) gene. Methylation of this gene has previously been associated with being small for gestational age (Liu et al., 2019).

While together these findings lend some support for an effect of the family therapy intervention on DNA methylation, a lack of prior evidence implicating these genes and gene regions, and the relatively small effect sizes observed which were not significant at genome‐wide corrected levels, means that these findings should be interpreted with caution. Further work is also needed to investigate whether these methylation changes remain stable over time, and how they might correlate with the psychological well‐being of the offspring.

DNA methylation‐based biological aging was not found to be different between the family therapy and the waitlist control groups. We used Horvath's clock as a marker of biological aging, which is a first‐generation epigenetic clock (Horvath, 2013) and one that has been extensively used in other studies. In older adult populations, these clocks have been shown to be highly predictive of mortality risk (Fransquet et al., 2019) and appear to vary in response to a range of exposures (Ryan et al., 2020). A meta‐analysis of nine studies showed that adults who had experienced childhood trauma had accelerated DNA methylation aging (Wolf et al., 2018). In children, Horvath's clock has shown to be highly correlated with chronical age (Fang et al., 2023), but few studies have investigated whether adverse exposures are associated with accelerated aging in childhood. In turn, it is not clear if accelerated aging early in life is a risk factor for adverse health outcomes. In a high‐risk cohort of 272 children aged 8–14 years, no association was found between maltreatment and accelerated epigenetic aging (Etzel et al., 2022), which aligns with our findings that family therapy had no beneficial effect on epigenetic aging. However, a study of 600 children aged 8–18 years found that more disadvantaged children had a faster pace of aging (Raffington et al., 2021). Other research has shown that some adverse exposures (e.g., smoking) were associated with accelerated aging in infancy, but others (including maternal stress and high blood pressure) were associated with reduced biological aging (Fransquet et al., 2023). These prior findings may help explain the lack of significant association observed in our study, as epigenetic aging appears to be highly dynamic in early life and influenced by a range of factors.

Strengths of our study relate to the randomized controlled design, with mothers and families randomly assigned to receiving first, either the family therapy intervention or the waitlist. This helps to ensure that any differences observed between the two groups are more likely to be due to the family therapy intervention itself. The family therapy program was delivered by KRCT staff who had received formal instruction by a certified trainer, and had an established relationship and rapport with the mothers. We collected blood samples both prior to the intervention and at the end of the 6‐month period, enabling interindividual changes in cortisol and DNA methylation patterns to be examined, and contrasted between the two groups. DNA methylation was measured using the Illumina EPIC (Illumina Inc.) array, enabling a hypothesis‐free examination of genes differently methylated in response to the family therapy.

There are a number of study limitations which should also be considered. Participants in the study needed to be registered in the KRCT database, and additional eligibility criteria included not having a major health condition, alcohol or substance abuse. This means our findings may not be generalizable to all individuals, with potentially the most traumatized and affected individuals excluded. The relatively small size of the study meant that we were only powered to detect very large effect sizes at epigenome‐wide corrected significance levels. This likely explains the lack of DNA methylation associations when accounting for multiple testing. We should also remain cautious about the nominally significant findings, which could be type 1 errors, and chance findings. The other major limitation of the study relates to the individual therapy mothers were able to access throughout the study. It was not ethically responsible to withhold individual therapy from mothers who sought additional support through KRCT. However, the proportion of mothers who underwent individual therapy was significantly higher in those assigned to the waitlist control group (53.1%), compared to the family‐intervention group (16.7%). This may have impacted the findings and made it more difficult to observe an effect of the family intervention. Finally, we only measured cortisol levels in the morning, at one time point, and this may not accurately reflect true cortisol levels in all participants. Release of cortisol follows a circadian rhythm, with levels increasing upon awakening, typically peaking within the first hour, and then declining across the day (Kirschbaum & Hellhammer, 1989). Capturing diurnal cortisol secretion throughout the day is therefore ideal to fully assess the cortisol levels of a given individual, but not always feasible, especially in studies involving children. This limitation may have influenced our findings, contributing to measurement error in the assessment of cortisol. However, we examined change in cortisol levels within an individual, rather than across individuals, and importantly, any measurement error is unlikely to be differential between the intervention and control groups. As such, this limitation should not have biased our results.

## 5 CONCLUSION

This is the first intergenerational study to investigate the effects of a tailored family‐based intervention on cortisol levels and DNA methylation patterns in children. Findings suggest the potential beneficial effects of the family intervention on supposed biological mechanisms involved in the intergenerational transmission of trauma. Further work is needed to investigate the longer term effects of this therapy.

## AUTHOR CONTRIBUTIONS

**Aung Zaw Zaw Phyo**: Investigation; formal analysis; visualization; writing – review and editing. **Dafina Arifaj Limani**: Methodology; data curation. **Feride Rushiti**: Resources; funding acquisition; methodology; conceptualization; supervision; writing – review and editing. **Line Hjort**: Conceptualization; methodology; funding acquisition; investigation; writing – review and editing. **Mimoza Salihu**: Methodology; data curation. **Nebahate Zekaj**: Methodology; data curation. **Peter Fransquet**: Methodology; supervision; writing – review and editing. **Qendresa Prapashtica**: Methodology; data curation. **Sara Helene Kaas‐Petersen**: Methodology; data curation; resources. **Sebahate Pacolli Krasniqi**: Supervision; resources; methodology; data curation. **Selvi Izeti Carkaxhiu**: Methodology; data curation; supervision; resources. **Shr‐Jie Wang**: Conceptualization; funding acquisition; writing – review and editing; methodology; supervision; resources; data curation. **Vesa Turjaka**: Methodology; data curation. **Vjosa Devaja Xhemaili**: Data curation; methodology. **Joanne Ryan**: Supervision; conceptualization; funding acquisition; writing – original draft; formal analysis; investigation.

## CONFLICT OF INTEREST STATEMENT

The authors declare no conflicts of interest.

### PEER REVIEW

The peer review history for this article is available at https://publons.com/publon/10.1002/brb3.70029.

6

**TABLE 1:** Characteristics of the mother–child dyads included in the study, according to intervention group.

	Family therapy (*n* = 30)	Waitlist control (*n* = 32)	*p*‐values^a^
*Children*			
Age in years, mean (standard deviation [SD])	10.8 (2.9)	9.9 (4.4)	.34
Sex, *n* (%)			
Males	15 (50%)	20 (62.5%)	.32
Females	15 (50%)	12 (37.5%)	
Living area, *n* (%)			
Rural	16 (53.3%)	19 (59.4%)	.82
Suburban	3 (10.0%)	2 (6.3%)	
Urban	11 (36.7%)	11 (34.4%)	
Birthweight (g), mean (SD)	3223.0 (638.1)	3317.7 (872.3)	.63
			
*Mother*			
Received individual counseling			
Yes	5 (16.7%)	17 (53.1%)	.003
No	25 (83.3%)	15 (46.9%)	
Post‐traumatic stress disorder (PTSD) during pregnancy			
Yes	16 (53.3%)	25 (78.1%)	.04
No	14 (46.7%)	7 (21.9%)	
Smoking during pregnancy			
Never	25 (83.3%)	18 (56.3%)	.02
Once a day	0 (0%)	1 (3.1%)	
Few times per day	2 (6.7%)	12 (37.5%)	
A lot each day	3 (10.0%)	1 (3.1%)	

^a^ *p*‐values from *t*‐tests or chi‐squared tests as appropriate.

**TABLE 2:** Top 10 differentially methylated probes^a^ between children receiving the family‐based intervention and the waitlist control group (*N* = 62).

Probe—CpG site	Closest gene	Delta *β*, %	Chrom.	Relation to Island	*p*‐value	Adjusted linear regression^b^	
						*Β* (95% CI)	*p*‐value
							
cg00392155	*CUL3*	+3.07%	chr2	N_Shore	3.01E−06	0.012 (−0.007, 0.032)	.21
cg11292453	*LPAR3*	−3.92%	chr1	Island	3.14E−06	−0.025 (−0.055, 0.004)	.09
cg09571353	*NKIRAS1*	+2.33%	chr3	OpenSea	3.19E−06	0.014 (0.001, 0.026)	**.04**
cg13149744	*ANKRD20B*	−1.58%	chr2	OpenSea	5.55E−06	−0.016 (−0.026, −0.005)	**.01**
cg19075928	*MKL2*	−1.36%	chr16	Island	5.65E−06	−0.010 (−0.021, 0.001)	.07
cg10429523	*RNF217‐AS1*	−2.56%	chr6	OpenSea	7.13E−06	−0.022 (−0.043, −0.000)	.05
cg25645178	*HM13*	+2.63%	chr20	Island	8.20E−06	0.026 (0.006, 0.045)	**.01**
cg16415121	*ORC3L;RARS2*	−1.97%	chr6	Island	9.60E−06	−0.006 (−0.019, 0.007)	.33
cg18741810		−1.53%	chr1	OpenSea	1.16E−05	−0.017 (−0.029, −0.005)	**.01**
cg17558817	*NCK2*	+3.31%	chr2	OpenSea	1.20E−05	0.032 (0.011, 0.053)	**.004**

*Note*: Bold values are significant.

^a^ Based on a minimum DNA methylation difference of at least 1%.

^b^ Adjusted for child sex, child age, child birthweight, living area, during pregnancy mothers’ post‐traumatic stress disorder (PTSD) status, and smoking status, whether mothers received individual therapy, array (for batch effect), cell counts (CD8+ T cells, CD4+ T cells, natural killer cells [NK], B lymphocytes [B cells], and neutrophils [Neu]), as well as baseline values for these probes.

**TABLE 3:** Top 10^a^ differentially methylated gene regions (DMR) between children receiving the family‐based intervention and the waitlist control group (*N* = 62).

Overlapping promoters	Chrom.	Start	End	Width	Number of CpGs	Max. ∆beta, %	Mean ∆beta, %	*p*‐value
*NHSL1*	chr6	1.39E+08	1.39E+08	529	5	16.2	9.3	.004
*AP001468.58, SPATC1L*	chr21	47604052	47605174	1123	9	10.2	6.4	.002
*RP1‐34H18.1*	chr12	77719732	77720159	428	5	6.3	4.3	.006
*MYCBPAP, RP11‐94C24.6*	chr17	48585216	48586147	932	14	9.3	3.6	4.35E−06
*IZUMO2*	chr19	50666238	50666558	321	6	7.0	3.4	.005
*GCC2*	chr2	1.09E+08	1.09E+08	608	3	5.7	3.2	.004
*LRRC27*	chr10	1.34E+08	1.34E+08	642	11	4.3	3.1	1.09E−05
*PKD1L2*	chr16	81253692	81254719	1028	9	6.3	2.8	.0006
*DPPA4*	chr3	1.09E+08	1.09E+08	527	5	3.3	2.5	.003
*SMAD5‐AS1*	chr5	1.35E+08	1.35E+08	296	4	4.1	2.2	.008

^a^ Based on the highest average DNA methylation difference.

## Supporting information


Supporting Information

Supporting Information

Supporting Information

## Data Availability

The data that support the findings of this study are available on request from the corresponding author.The data are not publicly available due to privacy or ethical restrictions.Due to the sensitivity nature, data cannot be publicly shared. However, requests to access deidentified data from this study can be made by contacting the authors.

## References

[brb370029-bib-0001] American Psychiatric Association . (2013). Diagnostic and statistical manual of mental disorders (DSM‐5) (5th ed.). American Psychiatric Association.

[brb370029-bib-0002] Amici, F. , Röder, S. , Kiess, W. , Borte, M. , Zenclussen, A. C. , Widdig, A. , & Herberth, G. (2022). Maternal stress, child behavior and the promotive role of older siblings. BMC Public Health, 22(1), 863. 10.1186/s12889-022-13261-2 35488325 PMC9055772

[brb370029-bib-0003] Aryee, M. J. , Jaffe, A. E. , Corrada‐Bravo, H. , Ladd‐Acosta, C. , Feinberg, A. P. , Hansen, K. D. , & Irizarry, R. A. (2014). Minfi: A flexible and comprehensive Bioconductor package for the analysis of Infinium DNA methylation microarrays. Bioinformatics, 30(10), 1363–1369. 10.1093/bioinformatics/btu049 24478339 PMC4016708

[brb370029-bib-0004] Association for Family Therapy and Systemic Practice . Accessed 7th December 2023. Retrieved from https://www.aft.org.uk/

[brb370029-bib-0005] Çay, M. (2018). Effect of increase in cortisol level due to stress in healthy young individuals on dynamic and static balance scores. Northern Clinics of Istanbul, 5(4), 295–301. 10.14744/nci.2017.42103 30859159 PMC6371989

[brb370029-bib-0006] Chu, D. A. , Williams, L. M. , Harris, A. W. F. , Bryant, R. A. , & Gatt, J. M. (2013). Early life trauma predicts self‐reported levels of depressive and anxiety symptoms in nonclinical community adults: Relative contributions of early life stressor types and adult trauma exposure. Journal of Psychiatric Research, 47(1), 23–32. 10.1016/j.jpsychires.2012.08.006 23020924

[brb370029-bib-0007] Cicchetti, D. , & Rogosch, F. A. (2001). Diverse patterns of neuroendocrine activity in maltreated children. Development and Psychopathology, 13(3), 677–693. 10.1017/s0954579401003145 11523854

[brb370029-bib-0008] Coussons‐Read, M. E. (2013). Effects of prenatal stress on pregnancy and human development: Mechanisms and pathways. Obstetric Medicine, 6(2), 52–57. 10.1177/1753495x12473751 27757157 PMC5052760

[brb370029-bib-0009] de Mendonça Filho, E. J. , Frechette, A. , Pokhvisneva, I. , Arcego, D. M. , Barth, B. , Tejada, C.‐A. V. , Sassi, R. , Wazana, A. , Atkinson, L. , Meaney, M. J. , & Silveira, P. P. (2022). Examining attachment, cortisol secretion, and cognitive neurodevelopment in preschoolers and its predictive value for telomere length at age seven. Frontiers in Behavioral Neuroscience, 16, 954977. 10.3389/fnbeh.2022.954977 36311861 PMC9606391

[brb370029-bib-0010] Di Costanzo, M. , De Paulis, N. , Capra, M. E. , & Biasucci, G. (2022). Nutrition during pregnancy and lactation: Epigenetic effects on infants' immune system in food allergy. Nutrients, 14(9), 1766. 10.3390/nu14091766 35565735 PMC9103859

[brb370029-bib-0011] Doom, J. R. , Cicchetti, D. , & Rogosch, F. A. (2014). Longitudinal patterns of cortisol regulation differ in maltreated and nonmaltreated children. Journal of the American Academy of Child & Adolescent Psychiatry, 53(11), 1206–1215. 10.1016/j.jaac.2014.08.006 25440310 PMC4254515

[brb370029-bib-0012] Etzel, L. , Hastings, W. J. , Hall, M. A. , Heim, C. M. , Meaney, M. J. , Noll, J. G. , O'donnell, K. J. , Pokhvisneva, I. , Rose, E. J. , Schreier, H. M. C. , Shenk, C. E. , & Shalev, I. (2022). Obesity and accelerated epigenetic aging in a high‐risk cohort of children. Scientific Reports, 12(1), 8328. 10.1038/s41598-022-11562-5 35585103 PMC9117197

[brb370029-bib-0013] Fang, F. , Zhou, L. , Perng, W. , Marsit, C. J. , Knight, A. K. , Cardenas, A. , Aung, M. T. , Hivert, M.‐F. , Aris, I. M. , Goodrich, J. M. , Smith, A. K. , Gaylord, A. , Fry, R. C. , Oken, E. , O'connor, G. , Ruden, D. M. , Trasande, L. , Herbstman, J. B. , Camargo, C A. , … Perera, F. (2023). Evaluation of pediatric epigenetic clocks across multiple tissues. Clinical Epigenetics, 15(1), 142. 10.1186/s13148-023-01552-3 37660147 PMC10475199

[brb370029-bib-0014] Fictorie, V. , Jonkman, C. , Visser, M. , Vandenbosch, M. , Steketee, M. , & Schuengel, C. (2022). Effectiveness of a high‐intensive trauma‐focused, family‐based therapy for youth exposed to family violence: Study protocol for a randomized controlled trial. Trials, 23(1), 46. 10.1186/s13063-021-05981-4 35039059 PMC8762952

[brb370029-bib-0015] Fransquet, P. D. , Hjort, L. , Rushiti, F. , Wang, S.‐J. , Krasniqi, S. P. , Çarkaxhiu, S. I. , Arifaj, D. , Xhemaili, V. D. , Salihu, M. , Leku, N. A. , & Ryan, J. (2022). DNA methylation in blood cells is associated with cortisol levels in offspring of mothers who had prenatal post‐traumatic stress disorder. Stress Health, 38(4), 755–766. 10.1002/smi.3131 35119793 PMC9790331

[brb370029-bib-0016] Fransquet, P. D. , Macdonald, J. A. , Ryan, J. , Greenwood, C. J. , & Olsson, C. A. (2023). Exploring perinatal biopsychosocial factors and epigenetic age in 1‐year‐old offspring. Epigenomics, 15(18), 927–939. 10.2217/epi-2023-0284 37905426

[brb370029-bib-0017] Fransquet, P. D. , Wrigglesworth, J. , Woods, R. L. , Ernst, M. E. , & Ryan, J. (2019). The epigenetic clock as a predictor of disease and mortality risk: A systematic review and meta‐analysis. Clinical Epigenetics, 11(1), 62. 10.1186/s13148-019-0656-7 30975202 PMC6458841

[brb370029-bib-0018] Goldstein, J. M. , Cohen, J. E. , Mareckova, K. , Holsen, L. , Whitfield‐Gabrieli, S. , Gilman, S. E. , Buka, S. L. , & Hornig, M. (2021). Impact of prenatal maternal cytokine exposure on sex differences in brain circuitry regulating stress in offspring 45 years later. Proceedings of the National Academy of Sciences of the United States of America, 118(15), e2014464118. 10.1073/pnas.2014464118 33876747 PMC8054010

[brb370029-bib-0019] Hjort, L. , Novakovic, B. , Grunnet, L. G. , Maple‐Brown, L. , Damm, P. , Desoye, G. , & Saffery, R. (2019). Diabetes in pregnancy and epigenetic mechanisms‐how the first 9 months from conception might affect the child's epigenome and later risk of disease. Lancet Diabetes & Endocrinology, 7(10), 796–806. 10.1016/s2213-8587(19)30078-6 31128973

[brb370029-bib-0020] Hjort, L. , Rushiti, F. , Wang, S.‐J. , Fransquet, P. , Krasniqi, S. , Çarkaxhiu, S. I. , Arifaj, D. , Xhemaili, V. D. , Salihu, M. , Leku, N. A. , & Ryan, J. (2021). Intergenerational effects of maternal post‐traumatic stress disorder on offspring epigenetic patterns and cortisol levels. Epigenomics, 13(12), 967–980. 10.2217/epi-2021-0015 33993712

[brb370029-bib-0021] Hogg, B. , Gardoki‐Souto, I. , Valiente‐Gómez, A. , Rosa, A. R. , Fortea, L. , Radua, J. , Amann, B L. , & Moreno‐Alcázar, A. (2023). Psychological trauma as a transdiagnostic risk factor for mental disorder: An umbrella meta‐analysis. European Archives of Psychiatry and Clinical Neuroscience, 273(2), 397–410. 10.1007/s00406-022-01495-5 36208317

[brb370029-bib-0022] Hoppen, T. H. , Meiser‐Stedman, R. , Kip, A. , Birkeland, M. S. , & Morina, N. (2024). The efficacy of psychological interventions for adult post‐traumatic stress disorder following exposure to single versus multiple traumatic events: A meta‐analysis of randomised controlled trials. Lancet Psychiatry, 11(2), 112–122. 10.1016/s2215-0366(23)00373-5 38219762

[brb370029-bib-0023] Horvath, S. (2013). DNA methylation age of human tissues and cell types. Genome Biology, 14(10), R115. 10.1186/gb-2013-14-10-r115 24138928 PMC4015143

[brb370029-bib-0024] Horvath, S. , Oshima, J. , Martin, G. M. , Lu, A. T. , Quach, A. , Cohen, H. , Felton, S. , Matsuyama, M. , Lowe, D. , Kabacik, S. , Wilson, J. G. , Reiner, A. P. , Maierhofer, A. , Flunkert, J. , Aviv, A. , Hou, L. , Baccarelli, A. A. , Li, Y. , Stewart, J. D. , … Raj, K. (2018). Epigenetic clock for skin and blood cells applied to Hutchinson Gilford Progeria Syndrome and ex vivo studies. Aging (Albany New York), 10(7), 1758–1775. 10.18632/aging.101508 PMC6075434 30048243

[brb370029-bib-0025] Johnson, N. M. , Hoffmann, A. R. , Behlen, J. C. , Lau, C. , Pendleton, D. , Harvey, N. , Shore, R. , Li, Y. , Chen, J. , Tian, Y. , & Zhang, R. (2021). Air pollution and children's health‐a review of adverse effects associated with prenatal exposure from fine to ultrafine particulate matter. Environmental Health and Preventive Medicine, 26(1), 72. 10.1186/s12199-021-00995-5 34253165 PMC8274666

[brb370029-bib-0026] Joubert, B. R. , Felix, J. F. , Yousefi, P. , Bakulski, K. M. , Just, A. C. , Breton, C. , Reese, S. E. , Markunas, C. A. , Richmond, R. C. , Xu, C.‐J. , Küpers, L. K. , Oh, S. S. , Hoyo, C. , Gruzieva, O. , Söderhäll, C. , Salas, L. A. , Baïz, N. , Zhang, H. , Lepeule, J. , … London, S. J. (2016). DNA methylation in newborns and maternal smoking in pregnancy: Genome‐wide consortium meta‐analysis. American Journal of Human Genetics, 98(4), 680–696. 10.1016/j.ajhg.2016.02.019 27040690 PMC4833289

[brb370029-bib-0027] Kirschbaum, C. , & Hellhammer, D. H. (1989). Salivary cortisol in psychobiological research: An overview. Neuropsychobiology, 22(3), 150–169. 10.1159/000118611 2485862

[brb370029-bib-0028] Kuo, P. X. , Saini, E. K. , Tengelitsch, E. , & Volling, B. L. (2019). Is one secure attachment enough? Infant cortisol reactivity and the security of infant‐mother and infant‐father attachments at the end of the first year. Attachment & Human Development, 21(5), 426–444. 10.1080/14616734.2019.1582595 30836833 PMC6779037

[brb370029-bib-0029] Küpers, L. K. , Xu, X. , Jankipersadsing, S. A. , Vaez, A. , La Bastide‐Van Gemert, S. , Scholtens, S. , Nolte, I. M. , Richmond, R. C. , Relton, C. L. , Felix, J. F. , Duijts, L. , Van Meurs, J. B. , Tiemeier, H. , Jaddoe, V. W. , Wang, X. , Corpeleijn, E. , & Snieder, H. (2015). DNA methylation mediates the effect of maternal smoking during pregnancy on birthweight of the offspring. International Journal of Epidemiology, 44(4), 1224–1237. 10.1093/ije/dyv048 25862628 PMC4588868

[brb370029-bib-0030] Lambertini, L. , Li, Q. , Ma, Y. , Zhang, W. , Hao, K. , Marsit, C. , Chen, J. , & Nomura, Y. (2019). Placental imprinted gene expression mediates the effects of maternal psychosocial stress during pregnancy on fetal growth. Journal of Developmental Origins of Health and Disease, 10(2), 196–205. 10.1017/s2040174418000545 30968809 PMC6786904

[brb370029-bib-0031] Liu, J. , Zhang, Z. , Xu, J. , Song, X. , Yuan, W. , Miao, M. , Liang, H. , & Du, J. (2019). Genome‐wide DNA methylation changes in placenta tissues associated with small for gestational age newborns; cohort study in the Chinese population. Epigenomics, 11(12), 1399–1412. 10.2217/epi-2019-0004 31596135

[brb370029-bib-0032] Løkhammer, S. , Stavrum, A.‐K. , Polushina, T. , Aas, M. , Ottesen, A A. , Andreassen, O. A. , Melle, I. , & Le Hellard, S. (2022). An epigenetic association analysis of childhood trauma in psychosis reveals possible overlap with methylation changes associated with PTSD. Translational Psychiatry, 12(1), 177. 10.1038/s41398-022-01936-8 35501310 PMC9061740

[brb370029-bib-0033] Lopes Cardozo, B. (2000). Mental health, social functioning, and attitudes of Kosovar Albanians following the war in Kosovo. JAMA, 284(5), 569–577. 10.1001/jama.284.5.569 10918702

[brb370029-bib-0034] Maksimovic, J. , Phipson, B. , & Oshlack, A. (2016). A cross‐package Bioconductor workflow for analysing methylation array data. F1000Research, 5, 1281. https://doi/org/10.12688/f1000research.8839.3 27347385 10.12688/f1000research.8839.1 PMC4916993

[brb370029-bib-0035] Mavranezouli, I. , Megnin‐Viggars, O. , Daly, C. , Dias, S. , Stockton, S. , Meiser‐Stedman, R. , Trickey, D. , & Pilling, S. (2020). Research review: Psychological and psychosocial treatments for children and young people with post‐traumatic stress disorder: A network meta‐analysis. Journalof Child Psychology and Psychiatry, 61(1), 18–29. 10.1111/jcpp.13094 31313834

[brb370029-bib-0036] Mcfarlane, A. C. (2010). The long‐term costs of traumatic stress: Intertwined physical and psychological consequences. World Psychiatry, 9(1), 3–10. 10.1002/j.2051-5545.2010.tb00254.x 20148146 PMC2816923

[brb370029-bib-0037] Meuret, A. E. , Trueba, A. F. , Abelson, J. L. , Liberzon, I. , Auchus, R. , Bhaskara, L. , Ritz, T. , & Rosenfield, D. (2015). High cortisol awakening response and cortisol levels moderate exposure‐based psychotherapy success. Psychoneuroendocrinology, 51, 331–340. 10.1016/j.psyneuen.2014.10.008 25462905

[brb370029-bib-0038] Monteagudo‐Sánchez, A. , Sánchez‐Delgado, M. , Mora, J. R. H. , Santamaría, N. T. , Gratacós, E. , Esteller, M. , De Heredia, M. L. , Nunes, V. , Choux, C. , Fauque, P. , De Nanclares, G. P. , Anton, L. , Elovitz, M. A. , Iglesias‐Platas, I. , & Monk, D. (2019). Differences in expression rather than methylation at placenta‐specific imprinted loci is associated with intrauterine growth restriction. Clinical Epigenetics, 11(1), 35. 10.1186/s13148-019-0630-4 30808399 PMC6390544

[brb370029-bib-0039] Motsan, S. , Yirmiya, K. , & Feldman, R. (2022). Chronic early trauma impairs emotion recognition and executive functions in youth; specifying biobehavioral precursors of risk and resilience. Development and Psychopathology, 34(4), 1339–1352. 10.1017/s0954579421000067 33779536

[brb370029-bib-0040] Nwanaji‐Enwerem, J. C. , Van Der Laan, L. , Kogut, K. , Eskenazi, B. , Holland, N. , Deardorff, J. , & Cardenas, A. (2021). Maternal adverse childhood experiences before pregnancy are associated with epigenetic aging changes in their children. Aging (Albany New York), 13(24), 25653–25669. https://doi/org/10.18632/aging.203776 10.18632/aging.203776 PMC8751604 34923483

[brb370029-bib-0041] Oberg, C. , & Sharma, H. (2023). Post‐traumatic stress disorder in unaccompanied refugee minors: Prevalence, contributing and protective factors, and effective interventions: A scoping review. Children (Basel), 10(6), 941. 10.3390/children10060941 37371174 PMC10296917

[brb370029-bib-0042] Oei, J. L (2020). Alcohol use in pregnancy and its impact on the mother and child. Addiction, 115(11), 2148–2163. 10.1111/add.15036 32149441

[brb370029-bib-0043] Perroud, N. , Rutembesa, E. , Paoloni‐Giacobino, A. , Mutabaruka, J. , Mutesa, L. , Stenz, L. , Malafosse, A. , & Karege, F. (2014). The Tutsi genocide and transgenerational transmission of maternal stress: Epigenetics and biology of the HPA axis. World Journal of Biological Psychiatry, 15(4), 334–345. 10.3109/15622975.2013.866693 24690014

[brb370029-bib-0044] Peters, T. J. , Buckley, M. J. , Statham, A. L. , Pidsley, R. , Samaras, K. , Lord, R. V. , Clark, S. J. , & Molloy, P. L. (2015). De novo identification of differentially methylated regions in the human genome. Epigenetics Chromatin, 8, 6. 10.1186/1756-8935-8-6 25972926 PMC4429355

[brb370029-bib-0045] Pidsley, R. , Zotenko, E. , Peters, T. J. , Lawrence, M. G. , Risbridger, G. P. , Molloy, P. , Van Djik, S. , Muhlhausler, B. , Stirzaker, C. , & Clark, S. J. (2016). Critical evaluation of the Illumina MethylationEPIC BeadChip microarray for whole‐genome DNA methylation profiling. Genome Biology, 17(1), 208. 10.1186/s13059-016-1066-1 27717381 PMC5055731

[brb370029-bib-0046] Radtke, K. M. , Ruf, M. , Gunter, H. M. , Dohrmann, K. , Schauer, M. , Meyer, A. , & Elbert, T. (2011). Transgenerational impact of intimate partner violence on methylation in the promoter of the glucocorticoid receptor. Translational Psychiatry, 1(7), e21. 10.1038/tp.2011.21 22832523 PMC3309516

[brb370029-bib-0047] Raffington, L. , Belsky, D. W. , Kothari, M. , Malanchini, M. , Tucker‐Drob, E. M. , & Harden, K. P. (2021). Socioeconomic disadvantage and the pace of biological aging in children. Pediatrics, 147(6), e2020024406. 10.1542/peds.2020-024406 34001641 PMC8785753

[brb370029-bib-0048] Ritchie, M. E. , Phipson, B. , Wu, D. , Hu, Y. , Law, C. W. , Shi, W. , & Smyth, G. K. (2015). Limma powers differential expression analyses for RNA‐sequencing and microarray studies. Nucleic Acids Research, 43(7), e47. 10.1093/nar/gkv007 25605792 PMC4402510

[brb370029-bib-0049] Ryan, J. , Chaudieu, I. , Ancelin, M.‐L. , & Saffery, R. (2016). Biological underpinnings of trauma and post‐traumatic stress disorder: Focusing on genetics and epigenetics. Epigenomics, 8(11), 1553–1569. 10.2217/epi-2016-0083 27686106

[brb370029-bib-0050] Ryan, J. , Mansell, T. , Fransquet, P. , & Saffery, R. (2017). Does maternal mental well‐being in pregnancy impact the early human epigenome? Epigenomics, 9(3), 313–332. 10.2217/epi-2016-0118 28140666

[brb370029-bib-0051] Ryan, J. , Wrigglesworth, J. , Loong, J. , Fransquet, P. D. , & Woods, R. L. (2020). A systematic review and meta‐analysis of environmental, lifestyle, and health factors associated with DNA methylation age. Journals of Gerontology, Series A: Biological Sciences and Medical Sciences, 75(3), 481–494. 10.1093/gerona/glz099 31001624 PMC7328212

[brb370029-bib-0052] Salas, L. A. , Koestler, D. C. , Butler, R. A. , Hansen, H. M. , Wiencke, J. K. , Kelsey, K. T. , & Christensen, B. C. (2018). An optimized library for reference‐based deconvolution of whole‐blood biospecimens assayed using the Illumina HumanMethylationEPIC BeadArray. Genome Biology, 19(1), 64. 10.1186/s13059-018-1448-7 29843789 PMC5975716

[brb370029-bib-0053] Suzuki, A. , Poon, L. , Papadopoulos, A. S. , Kumari, V. , & Cleare, A. J. (2014). Long term effects of childhood trauma on cortisol stress reactivity in adulthood and relationship to the occurrence of depression. Psychoneuroendocrinology, 50, 289–299. 10.1016/j.psyneuen.2014.09.007 25265282

[brb370029-bib-0054] Tarullo, A. R. , & Gunnar, M. R. (2006). Child maltreatment and the developing HPA axis. Hormones and Behavior, 50(4), 632–639. 10.1016/j.yhbeh.2006.06.010 16876168

[brb370029-bib-0055] Touleimat, N. , & Tost, J. (2012). Complete pipeline for Infinium(®) Human Methylation 450K BeadChip data processing using subset quantile normalization for accurate DNA methylation estimation. Epigenomics, 4(3), 325–341. 10.2217/epi.12.21 22690668

[brb370029-bib-0056] Wang, J. , & Zhou, W.‐H. (2021). Epigenetic clocks in the pediatric population: When and why they tick? Chinese Medical Journal (English), 134(24), 2901–2910. 10.1097/cm9.0000000000001723 PMC8710336 34520417

[brb370029-bib-0057] Wenzel, T. , Rushiti, F. , Aghani, F. , Diaconu, G. , Maxhuni, B. , & Zitterl, W. (2009). Suicidal ideation, post‐traumatic stress and suicide statistics in Kosovo. An analysis five years after the war. Suicidal ideation in Kosovo. Torture, 19(3), 238–247. 20065542

[brb370029-bib-0058] Wolf, E. J. , Maniates, H. , Nugent, N. , Maihofer, A. X. , Armstrong, D. , Ratanatharathorn, A. , Ashley‐Koch, A. E. , Garrett, M. , Kimbrel, N. A. , Lori, A. , Va Mid‐Atlantic Mirecc Workgroup , Aiello, A. E. , Baker, D. G. , Beckham, J. C. , Boks, M. P. , Galea, S. , Geuze, E. , Hauser, M. A. , Kessler, R. C. , … Logue, M. W. (2018). Traumatic stress and accelerated DNA methylation age: A meta‐analysis. Psychoneuroendocrinology, 92, 123–134. 10.1016/j.psyneuen.2017.12.007 29452766 PMC5924645

[brb370029-bib-0059] Yehuda, R. , & Lehrner, A. (2018). Intergenerational transmission of trauma effects: Putative role of epigenetic mechanisms. World Psychiatry, 17(3), 243–257. 10.1002/wps.20568 30192087 PMC6127768

[brb370029-bib-0060] Zajkowska, Z. , Gullett, N. , Walsh, A. , Zonca, V. , Pedersen, G. A. , Souza, L. , Kieling, C. , Fisher, H. L. , Kohrt, B. A. , & Mondelli, V. (2022). Cortisol and development of depression in adolescence and young adulthood—A systematic review and meta‐analysis. Psychoneuroendocrinology, 136, 105625. 10.1016/j.psyneuen.2021.105625 34920399 PMC8783058

[brb370029-bib-0061] Zhou, A. , & Ryan, J. (2023). Biological embedding of early‐life adversity and a scoping review of the evidence for intergenerational epigenetic transmission of stress and trauma in humans. Genes (Basel), 14(8), 1639. 10.3390/genes14081639 37628690 PMC10454883

[brb370029-bib-0062] Zietlow, A.‐L. , Nonnenmacher, N. , Reck, C. , Ditzen, B. , & Müller, M. (2019). Emotional stress during pregnancy—Associations with maternal anxiety disorders, infant cortisol reactivity, and mother‐child interaction at pre‐school age. Frontiers in Psychology, 10, 2179. 10.3389/fpsyg.2019.02179 31607996 PMC6773887

