# Supplementary material for: An epigenome‐wide study of a needs‐based family intervention for offspring of trauma‐exposed mothers in Kosovo
Source: Brain Behav. 2024 Sep 11;14(9):e70029. doi: 10.1002/brb3.70029 (PMC11391026; doi:10.1002/brb3.70029)
Supplement: Supplementary file 1 — Supporting Information [file BRB3-14-e70029-s002.docx]

**Supplementary Table S1.** The numbers of each probe type removed before further analysis.

| **Probe Type** | **Number of probes removed** |
| --- | --- |
| Failed probes (detection p-values > 0.01) | 11,614 |
| Probes with SNPs at CpG or SBE site | 30,020 |
| Sex Chromosome | 19,008 |
| Cross reactive | 39,087 |
| Total remaining for further analysis | 766,130 |

*Initial number of probes = 865,859

CpG, cytosine-phosphate-guanine; SBE, single base extension; SNP, single nucleotide polymorphism.

**Supplementary Table S2.** The unadjusted and fully adjusted linear regression results, for the association between the intervention vs. waitlist control group, and cortisol levels post-intervention

|  | **Children’s cortisol levels post-intervention** | |
| --- | --- | --- |
|  |  | |
|  | **Minimally adjusted model**^†^  Β (95% CI), p | **Fully Adjusted model**^‡^  Β (95% CI), p |
|  |  |  |
| Intervention vs waitlist control | -117.84 (-184.65, -51,02), p=0.001 | -124.72 (-197.37, -52,07), p=0.001 |

^†^Adjusted for only baseline cortisol levels

‡Adjusted for sex (boy/girl), age of the child (continuous), pregnancy PTSD (yes/no), maternal smoking during pregnancy (yes/no), individual therapy (yes/no) and baseline cortisol levels (continuous)

|  | **Prior to Intervention** | | | **Post Intervention** | | |
| --- | --- | --- | --- | --- | --- | --- |
|  | **Intervention Group**  Mean (SD) | **Control Group**  Mean (SD) | **p** | **Intervention Group**  Mean (SD) | **Control Group**  Mean (SD) | **p** |
|  |  |  |  |  |  |  |
| Horvath DNAm age | 13.25 (3.94) | 12.28 (5.36) | 0.43 | 13.66 (3.56) | 13.23 (5.61) | 0.72 |
| Horvath Skin Blood Clock DNAm age | 9.88 (2.94) | 9.29 (4.35) | 0.55 | 9.92 (3.00) | 9.26 (4.14) | 0.48 |
|  |  |  |  |  |  |  |
| Horvath Age Acceleration | -0.33 (2.50) | -0.40 (3.03) | 0.92 | 0.08 (1.83) | 0.61 (2.68) | 0.37 |

**Supplementary Table S5.** DNAm age and age acceleration in the intervention and waitlist control groups.

**Supplementary Table S6.** The difference in DNAm age between post-intervention and pre-intervention, in the two groups

|  | **Intervention**  Mean (SD) | **Control**  Mean (SD) | **P-values from t-tests** |
| --- | --- | --- | --- |
|  |  |  |  |
| Horvath DNAm age | 0.41 (1.86) | 0.98 (1.81) | 0.23 |
| Horvath Skin Blood Clock DNAm age | 0.04 (0.84) | -0.00 (0.86) | 0.85 |

**Supplementary Table S7.** The association between intervention group (intervention vs. waitlist control group) and biological aging

|  | **Crude model**  Β (95% CI), p | **Adjusted model^a^**  Β (95% CI), p |
| --- | --- | --- |
| **Pre-intervention (n = 60)** |  |  |
| Horvath DNAm age | 0.97 (-1.46, 3.40), p = 0.43 | 0.57 (-1.85, 2.98), p = 0.64 |
| Horvath Skin Blood Clock DNAm age | 0.58 (-1.34, 2.50), p = 0.55 | -0.12 (-1.28, 1.04), p = 0.84 |
| Horvath Age Acceleration | 0.07 (-1.37, 1.50), p = 0.92 | 0.57 (-1.85, 2.98), p = 0.64 |
|  |  |  |
| **Post-intervention (n = 62)** |  |  |
| Horvath DNAm age | 0.43 (-1.97, 2.84), p = 0.72 | -0.10 (-1.71, 1.51), p = 0.90 |
| Horvath Skin Blood Clock DNAm age | 0.65 (-1.20, 2.50), p = 0.48 | 0.12 (-1.02, 1.26), p = 0.84 |
| Horvath Age Acceleration | -0.53 (-1.70, 0.64), p = 0.37 | -0.10 (-1.71, 1.51), p = 0.90 |
|  |  |  |
| **Difference in DNAm age from post- to -pre- intervention (n = 60)** |  |  |
| Horvath DNAm age | -0.57 (-1.52, 0.37), p = 0.23 | -0.66 (-2.18, 0.86), p = 0.38 |
| Horvath Skin Blood Clock DNAm age | 0.04 (-0.40, 0.48), p = 0.85 | 0.56 (-0.06, 1.19), p = 0.07 |
|  |  |  |

^†^Linear regression model adjusted for sex (boy/girl), age of the child (continuous), pregnancy PTSD (yes/no), maternal smoking during pregnancy (yes/no), individual therapy (yes/no), array (for batch effect), cell counts (CD8+ T cells, CD4+ T cells, natural killer cells (NK), B lymphocytes (B cells), and neutrophils (Neu))
